# Sentiment analysis using Twitter data: a comparative application of lexicon- and machine-learning-based approach

**DOI:** 10.1007/s13278-023-01030-x

**Published:** 2023-02-09

**Authors:** Yuxing Qi, Zahratu Shabrina

**Affiliations:** 1 Centre for Urban Science and Progress, King’s College London, London, UK; 2 Department of Geography, King’s College, London, UK; 3 Regional Innovation, Graduate School, Universitas Padjadjaran, Bandung, Indonesia

## Introduction

Social media platform such as Twitter provides a space where users share their thoughts and opinion as well as connect, communicate, and contribute to certain topics using short, 140 characters posts, known as *tweets*. This can be done through texts, pictures, and videos, etc., and users can interact using likes, comments, and reposts buttons. According to Twitter (https://investor.twitterinc.com), the platform has more than 206 million daily active users in 2022, which is defined as the number of logged accounts that can be identified by the platform and where ads can be shown. As more people contribute to social media, the analysis of information available online can be used to reflect on the changes in people's perceptions, behavior, and psychology (Alamoodi et al. 2021). Hence, using Twitter data for sentiment analysis has become a popular trend. The growing interest in social media analysis has brought more attention to Natural Languages Processing (NLP) and Artificial Intelligence (AI) technologies related to text analysis.

Using text analysis, it is possible to determine the sentiments and attitudes of certain target groups. Much of the available literature focuses on texts in English but there is a growing interest in multilanguage analysis (Arun and Srinagesh 2020a; Dashtipour et al. 2016; Lo et al. 2017). Text analysis can be done by extracting subjective comments toward a certain topic using different sentiments such as Positive, Negative, and Neutral (Arun and Srinagesh 2020b). One of the topical interests would be related to the Coronavirus (Covid-19), which is a novel disease that was first discovered in late 2019. The rapid spread of Covid-19 worldwide has affected many countries, leading to changes in people’s lifestyles, such as wearing masks on public transportation and maintaining social distancing. Sentiment analysis can be implemented to social media data to explore changes in people’s behavior, emotions, and opinions such as by dividing the spread trend of Covid-19 into three stages and exploring people’s negative sentiments toward Covid-19 based on topic modeling and feature extraction (Boon-Itt and Skunkan 2020). Previous studies have retrieved tweets based on certain hashtags (#) used to categorize content based on certain topics such as “#stayathome” and “#socialdistancing” to measure their frequency (Saleh et al. 2021). Another study has used the Word2Vec technique and machine learning models, such as Naive Bayes, SVC, and Decision Tree, to explore the sentimental changes of students during the online learning process as various learning activities were moved online due to the pandemic (Mostafa 2021).

In this paper, we implement social media data analysis to explore sentiments toward Covid-19 in England. This paper aims to examine the sentiments of tweets using various methods including lexicon and machine learning approaches during the third lockdown period in England as a case study. Those who just started dealing with NLP should be able to use this paper to help select the appropriate method for their NLP analysis. Empirically, the case study also contributes to our understanding of the sentiments related to the UK national lockdown. In many countries, the implementation of policies and plans related to Covid-19 often sparked widespread discussion on Twitter. Tweet data can reflect the public sentiments on the Covid-19 pandemic, therefore providing an alternative source for guiding the government’s policies. The UK has experienced three national lockdowns since the outbreak of Covid-19, and people have expressed their opinions on Covid-19-related topics, such as social restrictions, vaccination plans, and school reopening, etc., all of which are worthy of exploring and analyzing. In addition, few existing studies focus on the UK or England, especially the change in people’s attitudes toward Covid-19 during the third lockdown.

## Sentiment analysis approaches

In applying sentiment analysis, the key process is classifying extracted data into sentiment polarities such as positive, neutral, and negative classes. A wide range of emotions can also be considered which is the focus of the emerging fields of affective computing and sentiment analysis (Cambria 2016). There are various ways to separate sentiments according to different research topics, for example in political debates, sentiments can be divided further into satisfied and angry (D’Andrea et al. 2015). Sentiment analysis with ambivalence handling can be incorporated to account for a finer-grained results and characterize emotions into such detailed categories such as anxiety, sadness, anger, excitement, and happiness (Wang et al. 2015, 2020).

Sentiment analysis is generally done to text data, although it can also be used to analyze data from devices that utilize audio- or audio-visual formats such as webcams to examine expression, body movement, or sounds known as multimodal sentiment analysis (Soleymani et al. 2017; Yang et al. 2022; Zhang et al. 2020). Multimodal sentiment analysis expands text-based analysis into something more complex that opens possibilities in the use of NLP for various purposes. Advancement of NLP is also rapidly growing driven by various research, for example in neural network (Kim 2014; Ray and Chakrabarti 2022). An example would be the implementation of Neurosymbolic AI that combines deep learning and symbolic reasoning, which is thought to be a promising method in NLP for understanding reasonings (Sarker et al. 2021). This indicates the wide possibilities of the direction of NLP research.

**Fig. 1:**
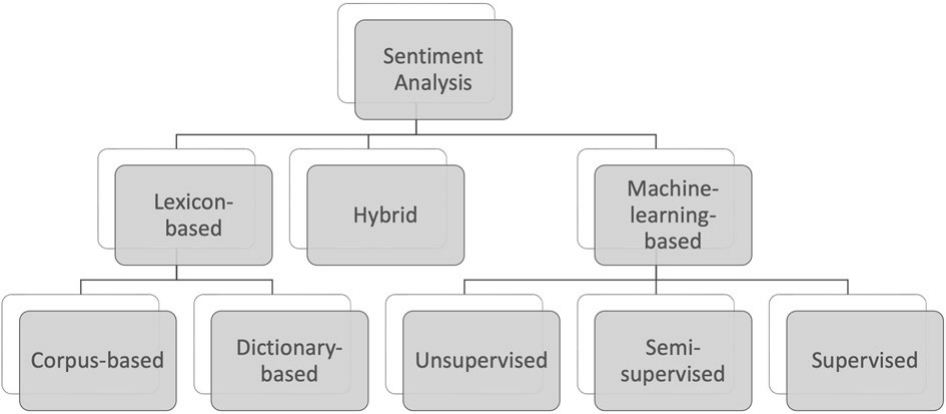
Sentiment analysis approaches

There are three main methods to detect and classify emotions expressed in text, which are lexicon-based, machine-learning-based approaches, and hybrid techniques. The lexicon-based approach uses the polarity of words, while the machine learning method sees texts as a classification problem and can be further divided into unsupervised, semi-supervised, and supervised learning (Aqlan et al. 2019). Figure 1 shows the classification of methods that can be used for sentiment analysis, and in practical applications, machine learning methods and lexicon-based methods could be used in combination.

When dealing with large text data such as those from Twitter, it is important to do the data pre-processing before starting the analysis. This includes replacing upper-case letters, removing useless words or links, expanding contractions, removing non-alphabetical characters or symbols, removing stop words, and removing duplicate datasets. Beyond the basic data cleaning, there is a further cleaning process that should be implemented as well including tokenization, stemming, lemmatization, and Part of Speech (POS) tagging. Tokenization splits texts into smaller units and turns them into a list of tokens. This helps to make it convenient to calculate the frequency of each word in the text and analyze their sentiment polarity. Stemming and lemmatization replace words with their root word. For example, the word “feeling” and “felt” can be mapped to their stem word: “feel” using stemming. Lemmatization, on the other hand, uses the context of the words. This can reduce the dimensionality and complexity of a bag of words, which also improves the efficiency of searching the word in the lexicon when applying the lexicon-based method. POS Tagging can automatically tag the POS of words in the text, such as nouns, verbs, and adjectives, etc., which is useful for feature selection and extraction (Usop et al. 2017).

### Lexicon-based approach

The core idea of the lexicon-based method is to (1) split the sentences into a bag of words, then (2) compare them with the words in the sentiment polarity lexicon and their related semantic relations, and (3) calculate the polarity score of the whole text. These methods can effectively determine whether the sentiment of the text is positive, negative, or neutral (Zahoor and Rohilla 2020). The lexicon-based approach performs the task of tagging words with semantic orientation either using dictionary-based or corpus-based approaches. The former is simpler, and we can determine the polarity score of words or phrases in the text using a sentiment dictionary with opinion words.

#### Lexicon-based approaches with built-in library

Examples of the most popular lexicon-based sentiment analysis models in Python are TextBlob and VADER. TextBlob is a Python library based on the Natural Language Toolkit (NLTK) that calculates the sentiment score for texts. An averaging technique is applied to each word to obtain the sentiment polarity scores for the entire text (Oyebode and Orji 2019). The words recorded in the TextBlob lexicon have their corresponding polarity score, subjectivity score, and intensity score. Additionally, there may be different records for the same word, so the sentiment score of the word is the average value of the polarity of all records containing them. The sentiment polarity scores produced are between [− 1, 1], in which − 1 refers to negative sentiment and + 1 refers to positive sentiment.

VADER (Valence Aware Dictionary and sEntiment Reasoner) is a lexicon and rule-based tool for sentiment analysis with a well-established sentiment lexicon (Hutto and Gilbert 2014). Compared to the TextBlob library, there are more corpora related to the language of social media, which may work better on a social media-type text that often contains non-formal language. From the results, the positive, negative, neutral, and compound values of tweets are presented, and the sentiment orientation is determined based on the compound score. There are several main steps of compound score calculation. Firstly, each word in the sentiment lexicon is given its corresponding scores of positive, negative, and neutral sentiments, ranging from − 4 to 4 from the most “negative” to the most “positive.” Heuristic rules are then applied when handling punctuation, capitalization, degree modifiers, contrastive conjunctions, and negations, which boosts the compound score of a sentence. The scores of all words in the text are standardized to (− 1, 1) using the formula below:

1 $$x=\frac{x}{\sqrt{{x}^{2}+a}}$$

where *x* represents the sum of Valence scores of sentiment words, and *α* is a normalization constant. The compound score is obtained by calculating the scores of all standardized lexicons in the range of − 1 (most negative) to 1 (most positive). The specific classification criteria for both TextBlob and VADER are shown in Table 1.

**Table 1:** Classification threshold of TextBlob and VADER

	TextBlob score	Sentiment orientation
TextBlob score	The polarity score > 0	Positive
	The polarity score < 0	Negative
	The polarity score = 0	Neutral
VADER compound score	The compound score > = 0.05	Positive
	The compound score < = −0.05	Negative
	The compound score > −0.05 and < 0.05	Neutral

#### Lexicon-based approach with SentiWordNet

**Fig. 2:**
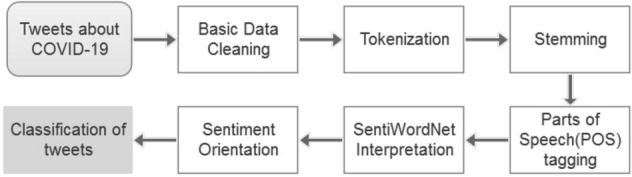
Process of SentiWordNet-based approaches

SentiWordNet is a lexical opinion resource that operates on the WordNet Database, which contains a set of lemmas with a synonymous interface called “synset” (Baccianella et al. 2010). Each synset corresponds to the positive and negative polarity scores. The value range of Pos(s) and Neg(s) is between 0 and 1. The process of SentiWordNet analysis is shown in Fig. 2.

There are several steps in applying the SentiWordNet-based approach. The first steps are data pre-processing including applying basic data cleaning, tokenization, stemming, and POS tagging. These steps can improve the time spent searching the words in the SentiWordNet database. For a given lemma that contains *n* meanings in the tweet, only the polarity score with the most common meaning is considered (the first one). The formula is as follows:

2 $$\mathrm{PosScore}=\mathrm{PosScore}1$$

3 $$\mathrm{NegScore}=\mathrm{NegScore}1$$

We can count the positive and negative terms in each tweet and calculate their sentiment polarity scores (Guerini et al. 2013). The sentiment score of each word or specific term in the SentiWordNet lexicon can be calculated by applying Eq. (4):

4 $$\mathrm{SynsetScore}=\mathrm{PosScore}-\mathrm{NegScore}$$

The SynsetScore then computes the absolute value of the maximum positive score and the maximum negative score of the word. For a term containing several synsets, the calculation is as follows:

5 $$\mathrm{TermScore}=\frac{\sum_{n=1}^{k}\mathrm{SynsetScore}(r)/r}{\sum_{n=1}^{k}1/r}$$

where *n* is a count number, the total score would be recorded as 0 if this term is not in SentiWordNet. The symbol *k* indicates how many synsets are contained in this term, and if there are negations in front of this term, then, this sentiment value is reserved. Finally, we can add the sentiment scores of all terms to get the sentiment score of the tweets using the formula below:

6 $$\mathrm{PosScore}\left(s\right)={\sum }_{i=1}^{m}\mathrm{TermScore}({T}_{i})$$

7 $$\mathrm{NegScore}\left(s\right)={\sum }_{i=1}^{n}\mathrm{TermScore}({T}_{i})$$

8 $$\mathrm{SentiScore}\left(s\right)=\mathrm{PosScore}\left(s\right)+\mathrm{NegScore}(s)$$

where *p* is a clean tweet with *m* positive terms and *n* negative terms. PosScore(*p*) is the final score of all the positive terms, while NegScore(*p*) represents the negative terms, and SentiScore(*s*) is the final sentiment score of tweets (Bonta et al. 2019).

### Machine learning approach

The machine learning approaches can construct classifiers to complete sentiment classification by extracting feature vectors, which mainly includes steps including data collecting and cleaning, extracting features, training data with the classifier, and analyzing results (Adwan et al. 2020). The dataset needs to be divided into a training and a test dataset using machine learning methods. The training sets aim to enable the classifier to learn the text features, and the test dataset evaluates the performance of the classifier.

**Fig. 3:**
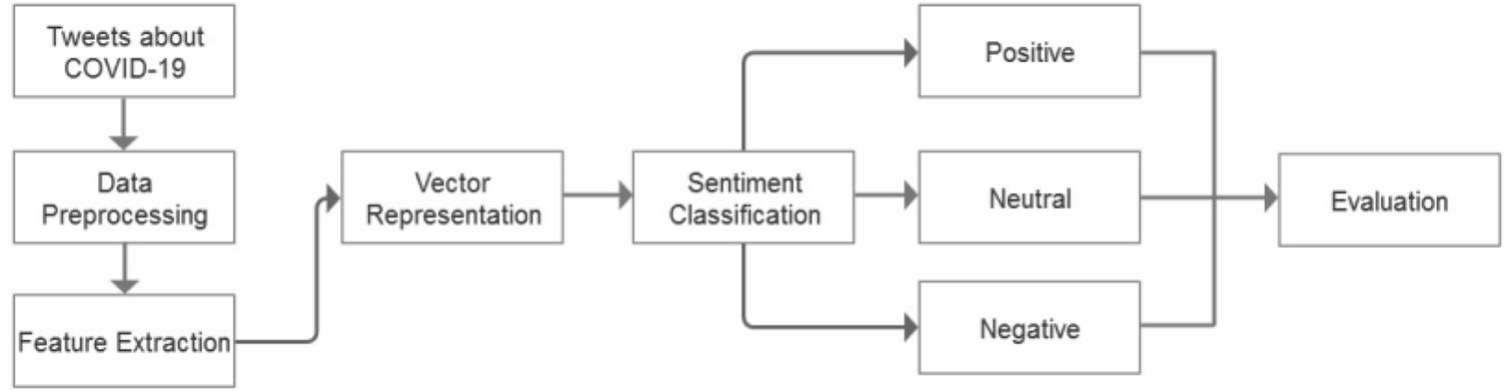
Main process of machine-learning-based approaches

The role of classifiers (e.g., Naïve Bayes classifier, Support Vector Machine, Logistic classifier, and Random Forest classifier.) is to classify text into different defined classes. As one of the most common methods for text classification, machine learning is widely used by researchers. In addition, the performance of the same classifier for different types of text may differ greatly, so the feature vectors of each type of text should be trained separately. To increase the robustness of the model, a two-stage support vector machine classifier can be used, which can effectively process the influence of noise data on classification (Barbosa and Feng 2010). In the subsequent process, it is necessary to vectorize the tweets data and divide the labeled tweets data into a training set (80%) and a test set (20%), and then, the sentiment labels can be predicted by training different classification models. The overall process is shown in Fig. 3 below:

#### Feature representation

The common methods of text feature representation can be divided into two categories: frequency-based embeddings (e.g., Count vector, Hashing Vectorizer, and TF–IDF) and pre-trained word embedding (e.g., Word2Vec, Glove, and Bert) (Naseem et al. 2021). In this paper, the following three feature representation models are mainly used:

1. *Bag of words* (*BoW*) converts textual data to numerical data with a fixed-length vector by counting the frequency of each word in tweets. In Python, CountVectorizer() works on calculating terms frequency, in which a sparse matrix of clean tokens is built.
2. *Term frequency–inverse document frequency* (*TF–IDF*) measures the relevance between a word and the entire text and evaluates the importance of the word in the tweet dataset. In Python, TfidfVectorizer() can obtain a TF–IDF matrix by calculating the product of the word frequency metric and inverse document frequency metric of each word from clean tweets.
3. *Word2Vec* generates a vector space according to all tweet corpus, and each word is represented in the form of a vector in this space. In the vector space, words with similar meanings will be closer together, so this method is more effective for dealing with semantic relations. In Python, the text embedding method can be implemented with the Word2Vec model in the Gensim library, and many different hyperparameters can be adjusted to optimize the word embedding model, such as setting various corpus (sentences), trying different training algorithms (skip-grams/sg), and adjusting the maximum distance between the current word and the predicted word in a sentence (window).

#### Classification models

Sentiment classification is the process of predicting users’ tweets as positive, negative, and neutral based on the feature representation of tweets. The classifiers in the supervised machine learning methods, such as a random forest, can classify and predict unlabeled text by training a large number of sentiment-labeled tweets. The classification models used in this paper are as follows:

##### Random forest

The results of the random forest algorithm are based on the prediction results of multiple decision trees, and the classification of new data points is determined by a voting mechanism (Breiman 2001). Increasing the number of trees can increase the accuracy of the results. There are several steps in applying random forest for text processing (Kamble and Itkikar 2018). First, we select *n* random tweet records from the dataset as the sample dataset and build a decision tree for each sample. We then get the predicted classification results of each decision tree. Then, we take the majority vote for each prediction of the decision trees. The sentiment orientation will be assigned to the category with the most votes. To evaluate the results, we can split the dataset into a training part to build the forest and a test part to calculate the error rate (al Amrani et al. 2018).

##### Multinomial Naïve Bayes

This model is based on the Naïve Bayes Theorem, which calculates the probability of multiple categories from many observations, and the category with the maximum probability is assigned to the text. Hence, the model can effectively solve the problem of text classification with multiple classes. The formula using Bayes Theorem to predict the category label based on text features (Kamble and Itkikar 2018) is as follows:

9 $$p\left(\frac{\mathrm{label}}{\mathrm{feature}}\right)=\frac{p(\mathrm{label})\times p(\mathrm{feature}/\mathrm{label})}{p(\mathrm{feature})}$$

where *p*(label) represents the prior probability of label *p*, and (feature/label) is the prior probability of the features with a given classifying label. To implement this technique, firstly, we calculate the prior probability for known category labels. Then, we obtain the likelihood probability with each feature for different categories and calculate the posterior probability with the formulas of the Bayes theorem. Lastly, we select the category with the highest probability as the label of the input tweet.

##### Support vector classification (SVC)

The purpose of this model is to determine linear separators in the vector space and facilitate the separation of different categories of input vector data. After the hyperplane is obtained, the extracted text features can be put into the classifier to predict the results. Additionally, the core idea is to find a line closest to the support vectors. The steps in implementing SVC include calculating the distance between the nearest support vectors, which is also called the margin, maximizing the margin to obtain an optimal hyperplane between support vectors from given data, and using this hyperplane as a decision boundary to segregate the support vectors.

#### Hyperparameters optimization

Hyperparameters can be considered as the settings of machine learning models, and they need to be tuned for ensuring better performance of models. There are many approaches to hyperparameter tuning, including Grid Search, Random Search, and automated hyperparameter optimization. In this study, Grid Search and Random Search are considered. The result may not be the global optimal solution of a classification model, but it is the optimal hyperparameters within the range of these grid values.

In applying Grid Search, we build a hyperparameter values grid, train a model with each combination of hyperparameter values, and evaluate every position of the grid. For Random Search, we build a grid of hyperparameter values and then, train a model with combinations randomly selected, which means not all the values can be tried. For this paper, this latter approach is more feasible because although the results of the Grid Search optimization method might be more accurate, it is inefficient and costs more time when compared with the random search approach.

## Data and methods

This paper focuses on tweets that were geotagged from the main UK cities during the third national Covid-19 lockdown. The cities are Greater London, Bristol, South Hampton, Birmingham, Manchester, Liverpool, Newcastle, Leeds, Sheffield, and Nottingham. Since the total number of tweets in each city is positively correlated with the urban population size and density, the number of tweets varies widely among these different cities. To collect more tweets to represent the perception of most people in England toward the Covid-19 pandemic, the selection criteria for major cities are based on the total population and density to improve the validity of the data (Jiang et al. 2016).

**Fig. 4:**
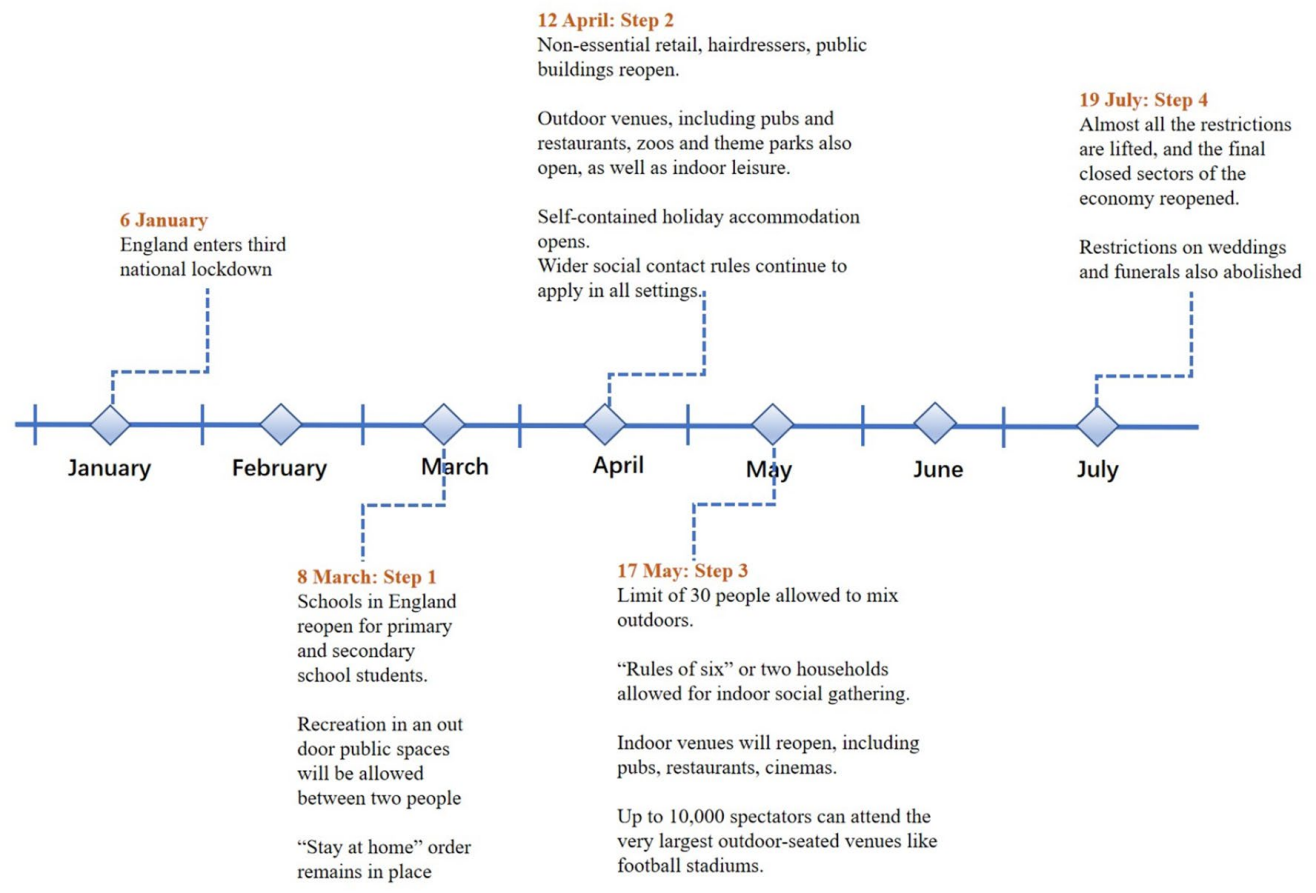
Detailed timeline of the third national lockdown in 2021

We divide the data collection time frame into three different stages of the third national lockdown in 2021. The timeline of the third national lockdown in England is from 6 January 2021 to 18 July 2021 as can be seen in Fig. 4. During this period, we selected several critical time points for research and analysis in stages according to the plan of lifting the lockdown in England, and the duration of each stage is about two months. The different stages are Stage 1 on January 6 until March 7, 2021, when England enters the third national lockdown, Stage 2 on March 8 until May 16, 2021, when the government implemented steps 1 and step 2 of lifting the lockdowns and Stage 3 on May 17 until July 18, 2021, when the government implemented step 3 of lifting the lockdown and easing most Covid-19 restrictions in the UK.

The tweets are extracted using Twint and Twitter Academic API, as these scraping tools can help facilitate the collection of tweets with geo-location, which helps in applying geographical analysis. However, users who are willing to disclose their geographic location when sending tweets only account for 1% of the total users (Sloan and Morgan 2015), and the location-sharing option is off by default. Therefore, the data collected by Twint and Twitter academic API are merged to obtain more tweets.

To filter the tweets related to Covid-19, we used keywords including “corona” or “covid” in the searching configuration of Twint or the query field of Twitter academic API, thus extracting the tweets and hashtags containing the search terms. In Twint, 1000 tweets can be fetched in each city per day, which avoids large bias in sentiment analysis due to uneven data distribution, but, in most cases, the number of tweets from a city for one day cannot reach this upper limit. Moreover, cities in the major cities list are used as a condition for filtering tweets from different geographic regions.

**Fig. 5:**
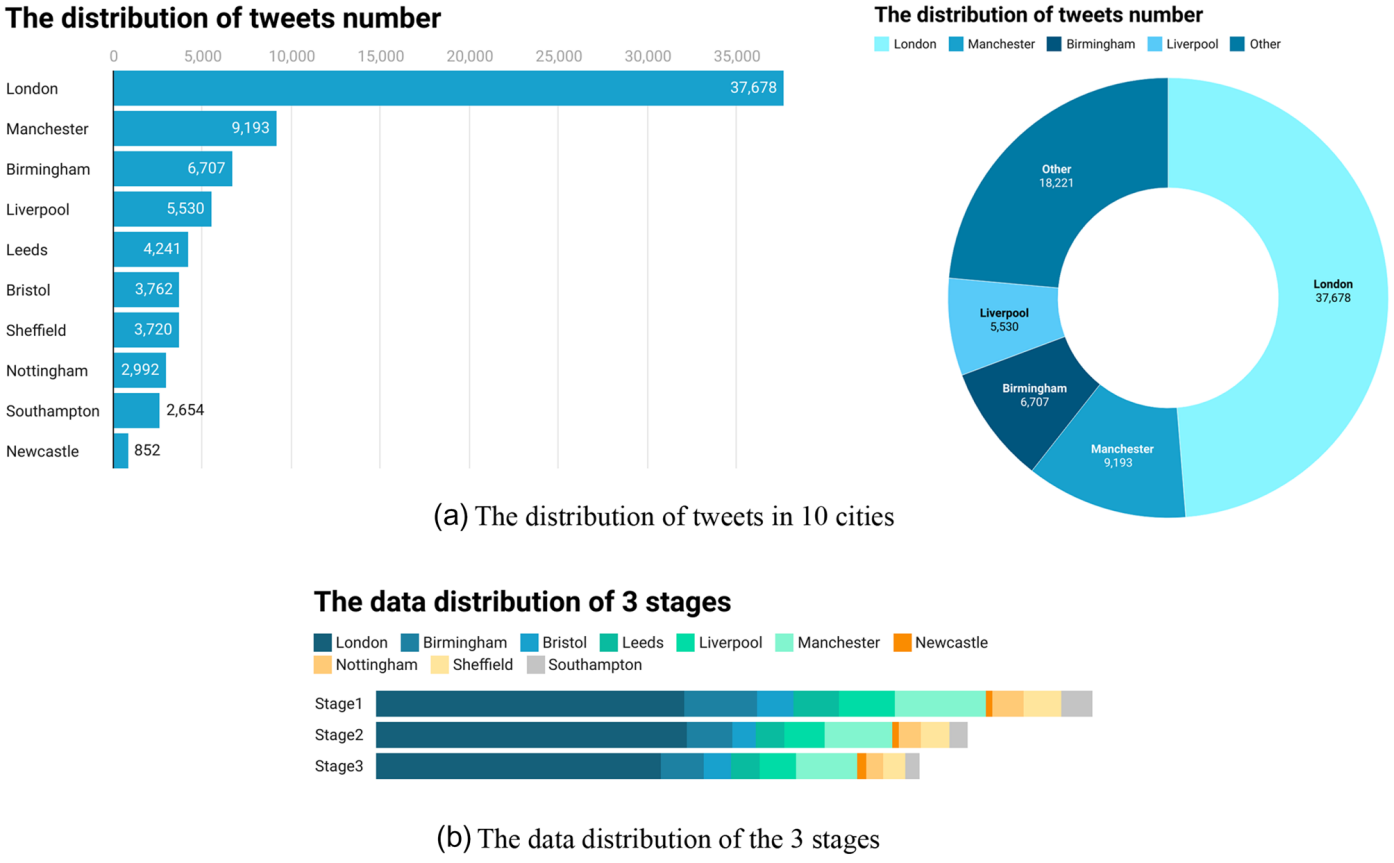
Distribution of collected tweets based on the selected cities and different stages

A total of 77,332 unique tweets were collected in three stages crawled from January 6 to July 18, 2021 (stage 1: 29,923; stage 2: 24,689; and stage 3: 22,720 tweets). The distribution of the number of tweets in each city is shown in Fig. 5a. Most of the tweets originate from London, Manchester, Birmingham, and Liverpool, and there are far more tweets in London (37,678) than in other cities. The number of tweets obtained in some cities, such as Newcastle, is much lower than the number of tweets in London, with only 852 tweets collected in six months. Figure 5 shows the distribution of data at each stage with the first stage having the most data while the third stage has the least amount of data. Additionally, at each stage, London has the largest proportion of data, with Newcastle having the least, linear to the total population and density of the area.

Since most raw tweets are unstructured and informal, which may affect the word polarity or text feature extraction, the data were pre-processed before sentiment analysis (Naseem et al. 2021). We implemented a basic data-cleaning process as follows:

1. Replacing upper-case letters to avoid recognizing the same word as different words because of capitalization.
2. Removing hashtags (#topic), mentioned usernames (@username), and all the links that start with “www,” “http,” and “https.” Removing stop words and short words (less than two characters). The stop words are mostly very common in the text but hardly contain any sentiment polarity. However, in sentiment analysis, “not” and “no” should not be listed as stop words, because removing these negations would change the real meaning of entire sentences.
3. Reducing repeated characters from some words. Some users will type repeated characters to express their strong emotions, so these words that are not in the lexicons should be converted into their corresponding correct words. For example: “sooooo goooood” becomes “so good.”
4. Expanding contractions in tweets such as “isn't” or “don't” as these will become meaningless letters or words after punctuations have been removed. Therefore, all contractions in the tweets are expanded into their formal forms, such as “isn’t” become “is not.”
5. Clearing all non-alphabetical characters or symbols including punctuation, numbers, and other special symbols that may affect the feature extraction of the text.
6. Removing duplicated or empty tweets and creating a clean dataset.
7. Converting emojis to their real meaning as many Twitter users use emojis in their tweets to express their sentiments and emotions. Hence, using the demojize() function in the emoji module of Python and transforming emojis into their true meaning may improve the accuracy of the sentiment analysis (Tao and Fang 2020).

In addition, for some sentiment analysis approaches, such as SentiWordNet-based analysis, further cleaning is essential, including stemming and POS Tagging.

In this study, strategies for text cleaning, polarity calculation, and sentiment classification model are designed and optimized using two different approaches to sentiment analysis: lexicon and machine-learning-based techniques. We then compared the results of the different methods and compare their output and prediction accuracy. The machine-learning-based approaches require labels with the tweets data, but the constraint is that it often takes too much time to manually annotate a large amount of data. Hence, 3000 tweets are randomly sampled in this paper, with the average number of tweets in each sentiment category of about 1000. To save the time spent on labeling, the classification results of the TextBlob or VADER method are used as the labels of the sample data (Naseem et al. 2021). We then manually check whether the classification of the VADER or TextBlob method is correct and modify it when necessary.

## Results and discussion

### Lexicon-based approach

**Fig. 6:**
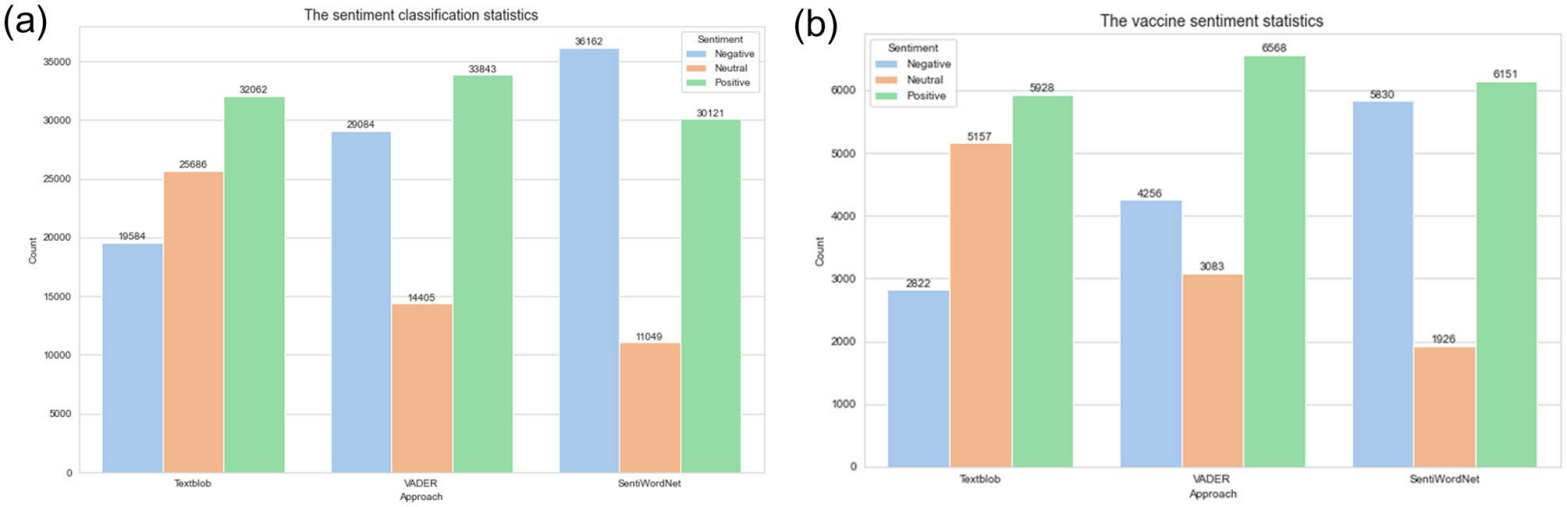
**a** Sentiment classification statistics, **b** vaccine sentiment statistics

From Fig. 6, the results obtained by TextBlob and VADER tools are similar, showing that positive sentiments appear more than negative sentiments. However, the number of neutral sentiments from the VADER method is lower. This might be because the VADER lexicon can efficiently handle the type of language used by social media users such as by considering the use of slang, Internet buzzwords, and abbreviations. On the other hand, TextBlob works better with formal language usage. Moreover, the results from the analysis using the SentiWordNet show a high proportion of negative sentiments. This might be due to some of the social media expressions of positive emotions that are not comprehensively recorded in the dictionary. Additionally, due to its limited coverage of domain-specific words, some words may be assigned wrong scores, which would cause a large deviation in sentiment scores. Only the most common meaning of each word is considered in SentiWordNet-based calculation; therefore, some large bias might occur. Consequently, the results of the VADER method are more convincing in this experiment. According to the comparison of public sentiment toward “Covid-19” and the “Covid-19 vaccine,” the classification results of all three approaches show that more people have positive sentiments than negative, indicating that most people expect the vaccine to have a good impact on Covid-19.

After using the lexicon-based approaches with TextBlob, VADER, and SentiWordNet-based methods, the sentiment scores and their classification results were obtained for each tweet. In this study, the three sentiment categories of positive, negative, and neutral sentiment correspond to 1, − 1, and 0, respectively, and we filter out the tweets in each city with their corresponding sentiment values (positive: 1, negative: − 1; and neutral: 0). The proportion of positive and negative sentiments in each city at each stage was calculated to compare how the sentiments change and to examine the differences in people’s perception of Covid-19 between these different cities.

**Fig. 7:**
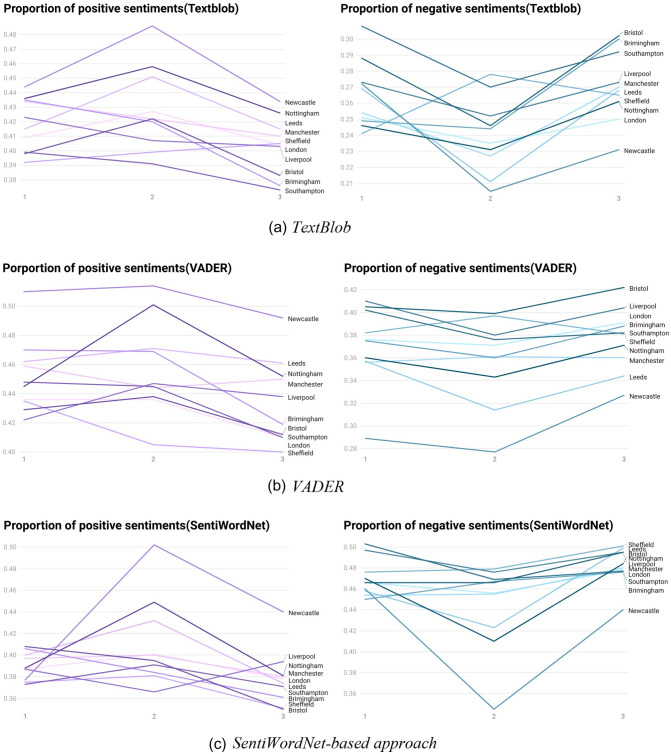
Results of the various lexicon-based approaches

Figure 7a indicates the results of using TextBlob in the three stages. In most cities in Fig. 7a, the proportion of positive sentiments at each stage is between 38 and 50%. Southampton and Manchester show a steady decline, while Sheffield is the only city where the proportion of positive sentiments increased in all three stages. Considering the entire period, Newcastle has the largest proportion of positive emotions, peaking at the second stage (about 50%), and Southampton was the lowest. For negative sentiments, the trend of Sheffield was different from other cities, which rise first and then fall. In addition, for most cities, the proportion of negative sentiments in the second stage is the lowest, and the proportion of negative sentiments in most cities is between 20 and 30%.

The results of VADER shown in Fig. 7b are similar to those of TextBlob. The proportion of positive sentiment in most cities is 40–50%, showing a trend of increasing first and then falling, except for Sheffield. Additionally, most of the negative sentiments account for between 30 and 40%. Moreover, the changes in the proportion of positive emotions in Manchester and Leeds are relatively flat, and the proportion of negative sentiments in Manchester also changes smoothly. However, Nottingham has a large change in positive sentiments at each stage, with a difference of about 6% between the highest and lowest values, and Newcastle has a wide range of negative sentiments proportion.

Based on the results of the SentiWordNet-based approach shown in Fig. 7c, the proportion of negative sentiments in each city is higher when compared with the previous two methods. Most of the negative sentiments are in the range of 40–50%, while the proportion of positive emotions is mostly between 36 and 46%. In terms of the trend of change, the percentage of Birmingham’s positive sentiment is declining, while the percentage of Liverpool’s positive sentiments trend is the opposite of other cities, which decreased first and then, increased.

Overall, according to the results of the three approaches, for most cities, the proportion of positive sentiments first rises and then, decreases. This is in contrast with the proportion of negative sentiments that decline from the first stage to the second stage and then, start to increase. The number of Covid-19 deaths and confirmed cases could be an indicator that can quantify the severity of the pandemic. Meanwhile, the increase in the number of people vaccinated with the Covid-19 vaccine can reduce the speed of the virus spreading among the population, thereby reducing the impact of the pandemic on people’s lives.

**Fig. 8:**
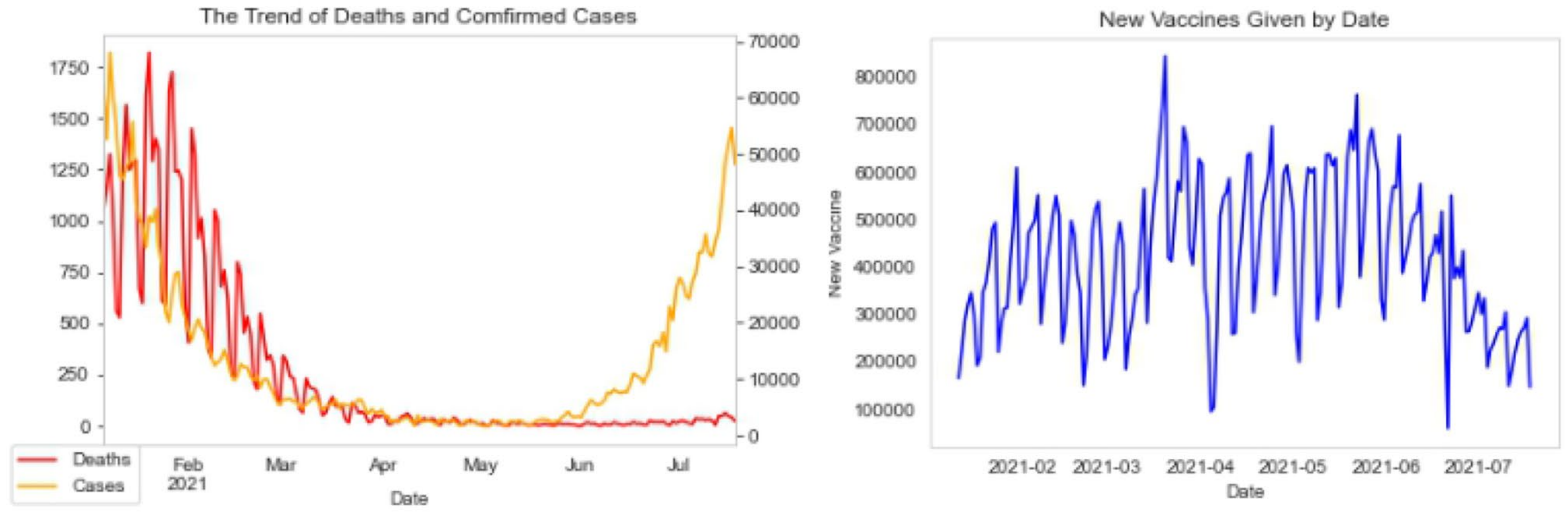
Trend of deaths, confirmed cases, and vaccines

Figure 8 shows the changes in the number of deaths and confirmed cases, and the number of new vaccines given. It shows that after peaking at the beginning of the third national lockdown, the number of deaths began to decline and became stable after April 2021. In addition, the number of newly confirmed cases in 2021 shows a downward trend from January to May but has increased significantly since June. Moreover, from the perspective of vaccination, the peak period of vaccination in 2021 is mainly in April and May, while after June, the vaccination volume drops greatly. Furthermore, combined with the previous results of sentiment analysis, from the first stage to the second stage, the positive sentiment proportion increases in most cities. This might be related to the improved situation of the Covid-19 pandemic as well as the increased number of vaccinations. However, there is a drop in positive sentiments from stage two to stage three, and the negative proportion increases. This might be due to the overall sentiment toward the vaccine’s protection rate and a large amount of new confirmed cases at the time. Overall, it might be that the public feels that the third lockdown policy and vaccination have not achieved the expected effect on the control of Covid-19 in England; hence, the number of negative sentiments has an upward trend after the second stage. More analysis is needed to explain the change in the sentiment trends more accurately.

### Machine-learning-based approach

In this paper, supervised learning approaches also need to be considered because unsupervised lexicon-based approaches cannot quantitatively analyze the results of sentiment classification. This part shows the classification performance of the three models (the proportion of the train dataset compared with the test dataset is 8:2) under different feature representation models (BoW, TF–IDF, and Word2Vec) and the optimization training on the models.

#### The hyperparameters of classification models

Each classification model needs to extract the text features of tweets and vectorize them before training, and the feature vectors of different forms may show different performances in the same classification model. Therefore, before the training of feature vectors, RandomizedSearchCV() is used to optimize the hyperparameters in the classifier. In the optimization process, the hyperparameters that are expected to be optimized can be selected with various options, and the result would be the optimal solution for the hyperparameters grid. Table 2(a) presents the optimal parameters of the random forest classifier, and Table 2(b) shows the optimal hyperparameters of the Multinomial Naive Bayes (MNB) classifier and the Support Vector Machine (SVC) classifier.

**Table 2:** Optimal hyperparameter based on the machine learning approach

Feature extraction	n_estimators	min_samples_split	min_samples_leaf	max_features	max_depth
*(a) The optimal hyperparameters of the random forest classifier*					
BoW	140	10	2	Auto	40
TF–IDF	120	5	2	Auto	40
Word2Vec	160	15	2	log2	30

Feature extraction	The optimal hyperparameters of MNB			The optimal hyperparameters of SVC	
	C	Kernel	Degree	alpha	fit_prior
*(b) Multinomial Naïve Bayes (MNB) and Support Vector Machine (SVC)*					
BoW	1	Linear	1	1.0	False
TF–IDF	10	Linear	3	0.5	False
Word2Vec	1000	Linear	2	10.0	False

#### The evaluation results of classifiers

These models classify all tweets into three categories, which are negative, positive, and neutral. The following Table 3 shows their performance with different feature representations.

**Table 3:** Model’s evaluation

Model	Category	Precision	Recall	F1-score	Accuracy
*Machine learning classifiers with BoW*					
Random Forest	Negative	0.95	0.25	0.40	0.7
	Neutral	0.80	0.70	0.74	
	Positive	0.64	0.94	0.76	
MultinomialNB	Negative	0.52	0.47	0.50	0.63
	Neutral	0.76	0.53	0.62	
	Positive	0.62	0.78	0.69	
SVC	Negative	0.6	0.59	0.59	0.71
	Neutral	0.70	0.79	0.74	
	Positive	0.79	0.73	0.76	
*Machine learning classifiers with TF–IDF*					
Random Forest	Negative	0.87	0.19	0.31	0.66
	Neutral	0.73	0.63	0.68	
	Positive	0.61	0.92	0.73	
MultinomialNB	Negative	0.50	0.48	0.49	0.62
	Neutral	0.70	0.49	0.58	
	Positive	0.64	0.77	0.70	
SVC	Negative	0.58	0.60	0.59	0.71
	Neutral	0.72	0.78	0.75	
	Positive	0.77	0.71	0.74	
*Machine learning classifiers with word embedding*					
Random Forest	Negative	0.47	0.12	0.19	0.53
	Neutral	0.61	0.47	0.53	
	Positive	0.51	0.82	0.63	
MultinomialNB	Negative	0.27	0.27	0.27	0.43
	Neutral	0.56	0.28	0.38	
	Positive	0.46	0.63	0.53	
SVC	Negative	0.75	0.06	0.11	0.56
	Neutral	0.71	0.50	0.59	
	Positive	0.52	0.92	0.66	

In this paper, Accuracy, Precision, and Recall are selected as evaluation indicators, measuring the performance of each classification model. Before calculating them, the values of the confusion matrix need to be known, and they are TP (True Positive), TN (True Negative), FP (False Positive), and FN (False Negative). Accuracy shows the proportion of the number of correct observations to the total observations using the formula below:

10 $$\mathrm{Accuracy}=\frac{\mathrm{TP}+\mathrm{TN}}{\mathrm{TP}+\mathrm{FP}+\mathrm{FN}+\mathrm{TN}}$$

Precision is the proportion of positive observations that correctly estimates the total number of positive predictions using the formula:

11 $$\mathrm{Precision}=\frac{\mathrm{TP}}{\mathrm{TP}+\mathrm{FP}}$$

Recall refers to the proportion of actual positive observations that are identified correctly calculated using:

12 $$\mathrm{Recall}=\frac{\mathrm{TP}}{\mathrm{TP}+\mathrm{FN}}$$

The F1 Score is a comprehensive evaluation and balance of precision and recall values, which can be calculated as follows:

13 $$\mathrm{F}1=\frac{2\times \mathrm{recall}\times \mathrm{precision}}{\mathrm{recall}+\mathrm{precision}}$$

According to the classification results of the three models, the performance of these classifiers for tweets with negative labels is poor, especially for the Random Forest Classifier, which has a low ability to recognize negative tweets, though the prediction precision is high. The reason for this may be that the labels are annotated manually, and unsupervised learning methods are different from the real sentiment expression of tweets. For the overall prediction, the SVC model has the best prediction ability with an accuracy of 0.71. Additionally, the F1 values of each label show that the SVC model has a good ability to classify the three categories of sentiments.

The accuracy of the three models is relatively high with the TF–IDF method, all above 60%. However, similar to the experimental results using the BoW feature representation, in Random Forest Classifier, the recall value of the negative category is very low, indicating that there are many negative tweets in the test dataset that have not been identified. This may be caused by the imbalanced distribution of data in each category, or the category contains some wrong data that would affect the training results. Moreover, these three models have the best predictive effect on the positive category, with an F1 score above 0.7. In summary, the performance of the SVC model is the best and the accuracy is higher than 70% in our study.

The prediction results of the three classifiers with Word2Vec are not as good as the previous two feature representation models, especially for the identification of negative sentiments. The reasons for the poor performance are that the Word2Vec embedding method needs to group semantically similar words, which requires a large amount of data, and it is difficult to extract sufficient text feature vectors from a small dataset. Moreover, compared with the Multinomial Naïve Bayes classifier, the SVC model and Random Forest classifier have better prediction performance, and their values of accuracy are 0.56 and 0.53, respectively.

## Conclusion

In conclusion, this paper extracts data regarding Covid-19 from people in the main cities of England on Twitter and separates it into three different stages. First, we perform data cleaning and use unsupervised lexicon-based approaches to classify the sentiment orientations of the tweets at each stage. Then, we apply the supervised machine learning approaches using a sample of annotated data to train the Random Forest classifier, Multinomial Naïve Bayes classifier, and SVC, respectively. From lexicon-based approaches, the three stages of public sentiment changes about the Covid-19 pandemic can be found. For most cities, the proportion of positive sentiments increases first and then drops, while the proportion of negative sentiments changed in a different direction. In addition, by analyzing the number of deaths and confirmed cases as well as vaccination situations, it could be concluded that the increase in confirmed cases and the decrease in vaccination volume might be the reason for the increase in negative sentiments, even though further research is needed to confirm this inference.

For supervised machine learning classifiers, the Random Search method is applied to optimize the hyperparameters of each model. The SVC results using BoW and TF–IDF feature models have the best performance, and their classification accuracy is as high as 71%. Due to the insufficiency of training data, the prediction accuracy of classifiers with the Word2Vec embedding method is low. Consequently, applying machine learning approaches to sentiment analysis can accurately extract text features without being restricted by lexicons.

It is important to note that this paper only collects the opinions of people in England on Twitter about Covid-19; thus, the result should be interpreted by considering this limitation. To obtain a more convincing conclusion, we can increase the data size by incorporating longer timeline, wider geographies, or by collecting data via other social media platforms while also considering the data protection policy. In addition, large-scale manually annotated datasets can be created for training machine learning models to improve their classification ability. Moreover, deep learning approaches can be used for model training, and this can be compared with different machine learning models. Furthermore, the Random Search method can only find the optimal parameters within a certain range, so exploring how to select model hyperparameters efficiently can further improve the stability of machine learning models. However, despite all the limitations, this study has provided contributions in advancing our understanding of the use of various NLP methods.

For lexicon-based approaches, the existing lexicon is modified to better fit the language habits of modern social media, improving the accuracy of this approach. Additionally, an annotated dataset can be created to compare the difference between predicted results and real results. Research on Covid-19 can be based on time series so that the changes in people’s attitudes and perceptions can be analyzed over some time. Moreover, further studies can combine the sentiment classification results with other factors such as deaths and vaccination rates and establish a regression model to analyze which factors contribute to the sentiment changes. Overall, the paper has showcased different methods of conducting sentiment analysis with SVC using BoW or TF–IDF outperformed the model accuracy overall.

## The codes of the project

The main codes of this project have uploaded to GitHub, and here is the link: https://github.com/Yuxing-Qi/Sentiment-analysis-using-Twitter-data.
